# A common marker of affect recognition dysfunction in the FTD spectrum of disorders

**DOI:** 10.1111/ene.16578

**Published:** 2024-12-04

**Authors:** Elisa Canu, Veronica Castelnovo, Edoardo Nicolò Aiello, Giulia De Luca, Elisa Sibilla, Fabiola Freri, Chiara Tripodi, Edoardo Gioele Spinelli, Giordano Cecchetti, Giuseppe Magnani, Francesca Caso, Paola Caroppo, Sara Prioni, Cristina Villa, Lucio Tremolizzo, Ildebrando Appollonio, Federico Verde, Nicola Ticozzi, Vincenzo Silani, Virginia E. Sturm, Katherine P. Rankin, Maria Luisa Gorno‐Tempini, Barbara Poletti, Massimo Filippi, Federica Agosta

**Affiliations:** ^1^ Neuroimaging Research Unit, Division of Neuroscience IRCCS San Raffaele Scientific Institute Milan Italy; ^2^ Neurology Unit IRCCS San Raffaele Scientific Institute Milan Italy; ^3^ Department of Neurology and Laboratory of Neuroscience IRCCS Istituto Auxologico Italiano Milan Italy; ^4^ Vita‐Salute San Raffaele University Milan Italy; ^5^ Fondazione IRCCS Istituto Neurologico Carlo Besta Unit of Neurology 5‐Neuropathology Milan Italy; ^6^ Fondazione IRCCS Istituto Neurologico Carlo Besta Clinical Neuropsychology Unit Milan Italy; ^7^ Neurology Unit, IRCCS “Fondazione San Gerardo” and School of Medicine and Surgery University of Milano‐Bicocca Monza Italy; ^8^ Department of Pathophysiology and Transplantation, “Dino Ferrari” Center Università degli Studi di Milano Milan Italy; ^9^ Memory and Aging Center University of California San Francisco San Francisco California USA; ^10^ Global Brain Health Institute University of California San Francisco San Francisco California USA; ^11^ Department of Oncology and Hemato‐Oncology Università degli Studi di Milano Milan Italy; ^12^ Neurophysiology Service IRCCS San Raffaele Scientific Institute Milan Italy; ^13^ Neurorehabilitation Unit IRCCS San Raffaele Scientific Institute Milan Italy

**Keywords:** CATS‐A, comprehensive affect testing system, emotion recognition, frontotemporal degeneration, FTD

## Abstract

**Background:**

Poor affect recognition is an early sign of frontotemporal dementia (FTD). Here, we applied the abbreviated version of the Comprehensive Affect Testing System (CATS‐A) battery to Italian FTD cases and healthy controls (HC) to provide cut‐offs of emotional dysfunction in the whole group and in different FTD clinical syndromes.

**Methods:**

One hundred thirty‐nine FTD patients (60 behavioural variant [bvFTD],13 semantic behavioural variant of FTD [sbvFTD], 28 progressive supranuclear palsy [PSP], 21 semantic [svPPA] and 17 nonfluent [nfvPPA] variants of primary progressive aphasia) and 116 HC were administered the CATS‐A, yielding an Affective Recognition Quotient (ARQ), which was used as outcome measure. Age‐ and education‐adjusted, regression‐based norms were derived in HC. In patients, the ARQ was assessed for its internal reliability, factorial validity and construct validity by testing its association with another social cognition paradigm, the Story‐Based Empath Task (SET). The diagnostic accuracy of the ARQ in discriminating patients from HC, genetic cases from HC and patient groups among each other was tested via ROC analyses.

**Results:**

In the whole FTD cohort, CATS‐A proved to be underpinned by a mono‐component factor (51.1%) and was internally consistent (McDonald's ω = 0.76). Moreover, the ARQ converged with the SET (*r*(122) = 0.50; *p* < 0.001) and optimally discriminated HC from both the whole cohort (AUC = 0.89) and each clinical syndrome (AUC range: 0.83–0.92). Conversely, CATS‐A subtests were able to distinguish patient groups.

**Conclusions:**

The ARQ score from the CATS‐A distinguishes FTD clinical syndromes from HC with high accuracy, making it an excellent tool for immediate use in clinical practice.

## INTRODUCTION

Social cognition refers to how people perceive, interpret and respond to social information, including others' thoughts, emotions and behaviours [1]. Among the subdomains of social cognition, social perception encompasses the recognition of familiar faces and the recognition of emotions from the face, body, voice and prosody [1]. Throughout the lifespan, people typically devote more time to observing faces than any other type of object [2], and even newborn infants show a preference for looking at face‐like patterns over other configurations, indicating that infants are born with some innate understanding of facial structure [3]. Moreover, the ability to comprehend another person's feelings has played a pivotal role in evolution. Rapidly recognizing emotions such as fear or disgust in others provides vital information that can enhance survival chances. The ability to link specific patterns of facial muscle contractions to distinct emotions is an inherent and universal feature in humans, unaffected by cultural differences [4].

Deficits in social cognition have been amply demonstrated in frontotemporal dementia (FTD), particularly in the behavioural variant of FTD (bvFTD), as an early marker of neurodegeneration [5, 6]. Impairment in social perception, particularly the misrecognition of negative facial emotions, has been extensively documented in bvFTD [7] and across the entire spectrum of FTD disorders [8], including progressive supranuclear palsy (PSP) [9], primary progressive aphasia (PPA) [10] and amyotrophic lateral sclerosis (ALS) [11].

The tools used to detect social perception deficits in FTD typically include paradigms for recognizing facial identity, identifying emotions from facial expressions, voice and prosody, matching similar emotional faces and interpreting emotions expressed through body postures [12, 13, 14]. Although these tools are commonly used, specific cut‐offs for the FTD spectrum and each clinical phenotype have not yet been established [13]. In this context, the Comprehensive Affect Testing System (CATS) [15] includes facial affect recognition (AR) tasks utilizing the static Ekman and Friesen faces. This battery is easy to administer in this population as it requires only brief and straightforward instructions, along with simple vocal or motor responses. Due to the length of the full version of the CATS, an abbreviated version of the battery (CATS‐A) has been implemented, which focuses on the two domains of AR and prosody recognition. CATS subtests have been successfully used in detecting AR failure in bvFTD [16, 17], as well as in other FTD variants [8, 11, 18].

Therefore, the present study aimed to assess the clinical usability of the AR subtests of the CATS‐A within the FTD spectrum by establishing specific AR cut‐off scores for this population in Italy. For this purpose, we administered the following CATS‐A subtests: affect discrimination (AD; 12 trials in which the patient is required to state whether two presented faces express the same or different emotions), name affect (NA; six trials in which the patient is required to select, among seven possible choices, the emotional label that best describes the emotion expressed by the target face), select affect (SA; six trials in which the patient is required to select, among five faces, the one that best reflects the target emotional label), match affect (MA; 12 trials in which the patient is required to select, among five possible facial affect pictures, the one expressing the same emotion of the face target) and the Three Faces Test (3FT; 24 trials in which the patient is required to select, among three facial affect pictures, the two faces that express the same emotion). Finally, we also administered a non‐AR subtest, the identity discrimination (ID; 12 trials in which the patient is required to state whether two presented faces represent the same or a different person).

## METHODS

A total of 343 patients with a suspected diagnosis of FTD‐related disorders were prospectively enrolled at five referral clinics in Lombardy, Italy, and referred to IRCCS San Raffaele Hospital in Milan between May 2017 and November 2022. Among them, we selected patients who: received a clinical diagnosis of an FTD clinical variant (i.e. probable bvFTD [6], probable nonfluent [nfvPPA] or semantic [svPPA] variants of PPA [19], PSP [20] or semantic behavioural variant of FTD [sbvFTD] [21]); performed clinical and neuropsychological assessments including an evaluation of AR with CATS‐A; and gave consent to be screened for known pathogenic mutations (i.e. C9orf72, GRN, MAPT, FUS, TREM2, TARDBP and SOD1). The final cohort included 139 FTD patients (60 bvFTD, 21 svPPA, 17 nfvPPA, 13 sbvFTD and 28 PSP). Seventy‐four patients (35 bvFTD, 10 nfvPPA, 10 svPPA, 14 PSP and 5 sbvFTD) also underwent lumbar puncture to exclude cerebrospinal fluid biomarker (CSF) profile suggestive of Alzheimer's disease pathology, as part of their diagnostic work‐up [22].

One hundred sixteen healthy controls (HC) were recruited by word of mouth among subjects unrelated to the patient population. They underwent a neurological and neuropsychological assessment which included the CATS‐A. All controls were recruited based on the following criteria: no family history of neurodegenerative diseases, and normal neurological and cognitive assessment.

Exclusion criteria for all subjects were as follows: medical illnesses or substance abuse that could interfere with cognitive functioning; any (other) major systemic, psychiatric or neurological illnesses; and (for patients only) other causes of focal or diffuse brain damage, including lacunae and extensive cerebrovascular disorders at a routine MRI.

### Standard protocol approvals, registrations and patient consents

Local ethical standards committee on human experimentation approved the study protocol and all participants provided written informed consent.

### Clinical evaluation

Clinical evaluations were performed by experienced neurologists. For all patients, disease severity was assessed using the CDR plus NACC FTLD [23] and independence with basic (ADL) and instrumental activities (IADL) of daily life [24, 25].

### Cognitive and behavioural assessment

In all patients, AR was evaluated using the CATS‐A [15], which investigates different aspects of emotion processing through the Ekman pictures of facial affect, depicting the six basic emotions. From this battery, we administered the following subtests: ID, AD, NA, SA, MA and 3FT. By following the original version of the CATS‐A [15], we obtained specific scores (i.e. number of correct answers) for each CATS‐A subdomain; furthermore, by summing the scores of all affect recognition subtests (all subtests except for CATS‐A ID), we obtained the total score of affect recognition quotient (ARQ).

The following cognitive functions were also investigated, as previously described: [26] global cognitive functioning, verbal and spatial memory, attention and executive functions, language, visuospatial abilities and behaviour. Full details are provided in the Appendix S1.

### Statistics

Patients and controls were compared on continuous sociodemographic, clinical and neuropsychological measures via linear models followed by Tukey‐corrected post hoc comparisons. When comparing neuropsychological measures, age, education and sex were entered as covariates. Chi‐square tests were employed for between‐group comparisons on categorical variables, followed by standardized residual‐based a posteriori decompositions (with cells yielding a *z*‐transformed residual ≥|2.87|, i.e. the critical value associated with the Bonferroni‐adjusted significance level, being deemed as significantly contributing to the *omnibus* effects).

Within the whole patient cohort, CATS‐A scores proved to distribute Normally—as indexed by skewness and kurtosis values <|1| and |3|, respectively [27], as well as by the absence of visual abnormalities in variable histograms and quantile‐quantile plots. Accordingly, linear model analyses were employed when addressing CATS‐A measures.

### Internal reliability, factorial validity and convergent validity

In the whole patient cohort, internal reliability and factorial validity of the CATS‐A were tested via McDonald's ω and principal component analysis (PCA), respectively. Accordingly, convergent validity of the ARQ was tested against the total score of the Story‐Based Empathy Task (SET) via a Pearson's correlation coefficient; within this analysis, in the aim of covarying for executive and receptive language deficits, Frontal Assessment Battery (FAB) and token test scores were partial led out.

### Affect recognition disease‐specific cut‐offs and diagnostics

Disease‐specific cut‐offs were derived for each CATS‐A subscore and the ARQ via a two‐step procedure. First, CATS‐A AD, NA and ARQ scores were adjusted for significant demographic confounders according to the normative equations previously derived by Castelnovo and colleagues [28]. In this study, the same norming approach [28, 29] was also employed to derive the adjustment equation for the CATS‐A ID (a subtest not included in the previous work [28]).

Second, in the aim of identifying disease‐specific cut‐offs, a series of receiver‐operating characteristics (ROC) analyses were run on either raw or demographically adjusted CATS‐A scores to discriminate both the whole FTD cohort and each patient group—that is the positive states—from HC. ROC analyses were also run to discriminate the genetic FTD cohort (g‐FTD) from HC, and the genetic bvFTD (g‐bvFTD) cases from HC.

Optimal cut‐offs were then identified at Youden's *J* statistic solely for those CATS‐A scores yielding an acceptable AUC value (i.e. ≥0.70) [29] within ROC analyses. These cut‐offs were computed for discriminating HC both from the whole FTD cohort and from each patient group, from g‐FTD, and from g‐bvFTD. Diagnostic metrics—that is sensitivity (Se), specificity (Sp), positive and negative predictive values (PPV; NPV) and likelihood ratios (LR+; LR‐)—were computed at the Youden's *J* statistic itself. Additionally, in order to evaluate the overall diagnostic quality of these cut‐offs, the Summary Utility Index (SUI) was computed as the following: (Se*PPV) + (Sp*NPV); with values <0.97 suggesting an overall ‘poor’ diagnostic performance, values ≥0.98 suggesting an overall ‘acceptable’ (‘adequate’) diagnostic performance, and values ≥1.28 suggesting an overall ‘good’ diagnostic performance [30].

### Case–case discrimination

In order to determine whether the CATS‐A was able to discriminate between different FTD phenotypes, its total and subtest scores were entered into a series of linear models addressing each patient group as the predictor. In the aim of these analyses, CATS‐A scores were demographically adjusted whenever necessary. Based on the significant different CATS‐A scores, ROC analyses were performed on either raw or demographically adjusted CATS‐A scores for discriminating patient groups among each other, the g‐FTD from sporadic FTD (s‐FTD), and the g‐bvFTD from sporadic bvFTD (s‐bvFTD). Optimal cut‐offs were then identified only for those CATS‐A scores yielding an acceptable AUC value (i.e. ≥0.70), as reported above.

### Software

Analyses were run via IBM® SPSS® Statistics 29 (IBM Corp., 2023), jamovi 2.3 (https://www.jamovi.org/) and R 4.1 (https://cran.r‐project.org/). Missing data were excluded pairwise. The significance threshold was set at *α* = 0.05 and Bonferroni‐corrected whenever adequate.

## RESULTS

Table 1 summarizes participants' demographic, clinical and neuropsychological measures.

**TABLE 1:** Demographic, clinical and neuropsychological measures of the sample.

	bvFTD	sbvFTD	nfvPPA	svPPA	PSP	HC	*p*
*N*	60	13	17	21	28	116	
Sex (M)	62%	77%	35%	48%	29%	39%	0.004^a^
Age (years)	65 ± 8.1* (36–79)	61.5 ± 9.7* (48–77)	67.4 ± 10.3 (51–83)	64.1 ± 9.4* (42–81)	71.2 ± 6.7 (60–85)	63.6 ± 8.3* (40–84)	<0.001
Education (years)	10.5 ± 3.3 (2–19)^#^	9.9 ± 3.1 (5–13)§	11.4 ± 5.5 (5–22)	12.7 ± 4.2 (5–18)	9.3 ± 4.9 (5–23)^#^	12.8 ± 4.2 (5–27)	<0.001
Disease duration (months)	38.7 ± 28.1 (4–129)	37.5 ± 24.5 (13–102)	31.2 ± 17.7 (10–73)	55.1 ± 50.5 (11–242)	32.7 ± 12.9 (11–70)	–	0.229
Genetics (*N*)							
*C9orf72*	4	–	–	–	–	–	
*MAPT*	2	1	–	1	–	–	
*C9orf72 +* MAPT	1						
*FUS*	1	–	–	–	–	–	
*GRN*	9	1	3	1	1	–	
*TREM2*	1	–	–	–	–	–	
MMSE	23.4 ± 5.3# (6–30)	25.6 ± 3.9 (18–30)	25.1 ± 6.3# (5–30)	22 ± 6.8#* (5–30)	25 ± 4.1# (16–30)	29.2 ± 1 (26–30)	<0.001
FAB	11.2 ± 3.7 (1–17)	13.2 ± 3 (5–16)	12.2 ± 2.9 (7–17)	12.6 ± 3.7 (3–17)	10.3 ± 4.3 (3–18)	–	0.149
Memory							
RAVLT, immediate recall	24.8 ± 10# (0–43)	28.2 ± 10.3# (14–44)	30.3 ± 13.1# (4–49)	22.1 ± 10.2#$ (7–43)	25.5 ± 10.7# (7–46)	47.1 ± 8.6 (20–67)	<0.001
RAVLT, delayed recall	3.4 ± 3# (0–9)	3.9 ± 3.1# (0–10)	6.5 ± 3.5#§ (0–12)	2.6 ± 3.2#$ (0–10)	4.2 ± 3.4# (0–12)	10.2 ± 2.7 (4–15)	<0.001
Digit span, forward	4.9 ± 1.2# (2–7)	5.4 ± 1 (3–7)	4.2 ± 0.8# (3–6)	4.9 ± 1.3# (2–7)	4.4 ± 1.2# (2–7)	6 ± 1 (4–9)	<0.001
Spatial span, forward	3.8 ± 1.4# (0–7)	4.7 ± 1.7 (0–7)	4.1 ± 1.2# (2–6)	4.5 ± 1.2 (2–7)	3.8 ± 1.3# (1–6)	5.2 ± 1.1 (3–7)	<0.001
ROCF, delayed recall	7.2 ± 5.9# (0–27.5)	9.3 ± 5.9# (0–18.5)	9.9 ± 4.2# (4–16.5)	10 ± 7.8# (0–25)	7.6 ± 5.9# (0–20.5)	16.5 ± 6.6 (6.5–34)	<0.001
Attention and executive functions							
Digit span, backward	3.2 ± 1.3# (0–5)	4.1 ± 0.9*$ (2–5)	2.3 ± 1.3# (0–4)	3.2 ± 1.3# (0–5)	2.5 ± 1.5# (0–6)	4.7 ± 1.2 (2–8)	<0.001
Attentive matrices	39 ± 12.5#* (4–60)	48.3 ± 6.6*§ (35–59)	38.3 ± 12# (11–54)	42.7 ± 12.6#* (13–59)	30.1 ± 13.1# (10–58)	52.2 ± 6.5 (27–60)	<0.001
TMT, B‐A	158.2 ± 125.5# (14–618)	105.7 ± 41.9 (43–181)	120.7 ± 49.1 (55–220)	113.3 ± 50.4 (26–224)	180.8 ± 91.5# (59–379)	64.6 ± 38.2 (0–209.7)	<0.001
MCST, categories	2.4 ± 1.7# (0–6)	3.7 ± 2.3 (0–6)	3.1 ± 2.1 (0–6)	4.4 ± 1.6§ (2–6)	3.1 ± 2 (0–6)	4.4 ± 1.3 (1–6)	<0.001
MCST, perseverations	13.9 ± 9.6# (0–44)	5.7 ± 7.2§ (0–20)	11.9 ± 12.5# (0–37)	5.7 ± 8.1§ (0–34)	9.5 ± 10 (0–47)	3.7 ± 3.3 (0–16)	<0.001
RCPM	20.5 ± 7.7# (4–35)	25 ± 7.2# (5–34)	24.3 ± 7.2# (6–34)	24.6 ± 7.4# (10–36)	20 ± 7.3# (5–34)	31.5 ± 3.6 (17–36)	<0.001
Social cognition							
ET, global score	9.9 ± 3.3 (5–18)	8.7 ± 2.5 (5–13)	9.7 ± 4.7 (0–16)	10.5 ± 4.8 (2–18)	10.3 ± 3.7 (5–17)	–	0.593
SET‐IA	3.5 ± 1.5 (1–6)	2.5 ± 1.4 (0–5)	3.2 ± 1.7 (0–6)	3.4 ± 1.9 (0–6)	3.3 ± 1.6 (1–6)	–	0.475
SET‐CI	3.3 ± 1.5 (0–6)	3.3 ± 1.6 (1–5)	3.4 ± 1.6 (0–6)	4.1 ± 1.6 (1–6)	3.3 ± 1.8 (0–6)	–	0.811
SET‐EA	3.1 ± 1.3 (1–6)	2.8 ± 1.5 (1–6)	3.3 ± 1.9 (0–6)	3 ± 2 (0–6)	3.6 ± 1.3 (2–6)	–	0.174
CATS‐A ID	9.2 ± 2.2# (5–12)	11.2 ± 1.1§ (9–12)	10.4 ± 3.1 (0–12)	10.5 ± 1.9 (5–12)	9.6 ± 2.2# (6–12)	11.6 ± 0.7 (8–12)	<0.001
CATS‐A AD	9.4 ± 1.9# (6–12)	11.2 ± 1§$ (9–12)	9.5 ± 2.9# (0–12)	10.4 ± 1.9 (5–12)	9.8 ± 1.9 (6–12)	11.1 ± 1 (7–12)	<0.001
CATS‐A NA	2.7 ± 1.5# (0–6)	2.8 ± 1.2# (1–5)	3.1 ± 1.6# (0–6)	3 ± 1.8# (0–6)	2.9 ± 1.3# (1–6)	4.6 ± 1.1 (2–6)	<0.001
CATS‐A SA	3.5 ± 1.4# (0–6)	3.2 ± 0.9# (2–5)	4 ± 1.8# (0–6)	4.2 ± 1.3# (2–6)	3.9 ± 1.5# (1–6)	5.5 ± 0.7 (3–6)	<0.001
CATS‐A MA	6 ± 2# (1–10)	5.7 ± 0.8# (4–7)	5.2 ± 2.8# (0–9)	7.2 ± 1.8# (4–11)	6 ± 2.6# (0–10)	8.8 ± 1.9 (2–12)	<0.001
CATS‐A 3FT	9.8 ± 2.9# (4–18)	10.3 ± 2.5# (7–16)	8 ± 3.6#* (0–14)	11.5 ± 2.7#$ (8–19)	10.8 ± 2.9# (6–18)	13.9 ± 3.3 (6–22)	<0.001
CATS‐A ARQ	31.4 ± 6.8# (15–48)	33.2 ± 4.1# (27–43)	29.9 ± 10.2# (0–44)	36.4 ± 6.2# (28–53)	33.4 ± 7.1# (22–46)	43.8 ± 5.6 (22–57)	<0.001
Language							
Phonemic fluency	16.4 ± 10.7# (0–47)	17.3 ± 6.8# (7–33)	12.1 ± 8.5# (1–25)	18.6 ± 10.1# (0–31)	12.3 ± 9.6# (1–35)	37.8 ± 9.9 (12–64)	<0.001
Semantic fluency	21.5 ± 11.2# (1–58)	21.5 ± 11.4# (0–36)	23.8 ± 11.1# (0–37)	12.4 ± 8.2#*§$ (0–33)	21 ± 7.5# (7–36)	48 ± 10.5 (25–71)	<0.001
CaGi‐Naming	–	33.5 ± 13.6 (0–46)	42.4 ± 11.3 (7–48)	21.1 ± 14$ (0–48)	–	–	<0.001
CaGi‐WPM	–	43.9 ± 5.6 (31–48)	47.2 ± 2.1 (41–48)	41.1 ± 7.2$ (24–48)	–	–	0.016
PPT	–	39.9 ± 6 (27–47)	46.4 ± 6.8 (29–52)	37.9 ± 10.9$ (7–51)	–	–	0.021
Token Test	27.3 ± 7.1# (5–36)	27.5 ± 9# (4–35)	24.5 ± 8.5# (0–32)	24.6 ± 10.2# (3–36)	26.3 ± 5.6# (13–35)	33.9 ± 1.9 (27–36)	<0.001
Visuospatial abilities							
CDT	5.6 ± 3.6 (0–10)	6.2 ± 3.9 (0–10)	6.5 ± 3.7 (0–10)	5.1 ± 4 (0–10)	4.7 ± 3.8 (0–10)	–	0.665
ROCF, copy	23.7 ± 11.2# (0–60)	30.3 ± 3.1* (25–35)	26.3 ± 7.8 (6–34)	28.5 ± 6.1* (14–35)	19.5 ± 7.5# (6.5–34)	16.5 ± 6.6 (6.5–34)	<0.001
FBI, Section A	14.7 ± 6.5 (0–27)	13.2 ± 4.7 (8–20)	7.9 ± 5.7§ (0–20)	12.2 ± 8.4 (2–28)	12.3 ± 7.3 (0–31)	–	0.032
FBI, Section B	8.5 ± 6.3 (0–24)	8.3 ± 5.9 (2–17)	3.2 ± 4.5§ (0–16)	6.6 ± 5.9 (0–20)	5.6 ± 5.4 (0–21)	–	0.024
FBI, Total	23.4 ± 11.2 (0–51)	21.5 ± 9.4 (11–37)	11.1 ± 9.6§ (0–36)	18 ± 12.2 (6–40)	17.2 ± 10.1 (1–46)	–	0.003

*Note*: Comparisons on continuous measures were run via linear models followed by Tukey‐corrected, post hoc comparisons. Comparisons on neuropsychological measures were run by entering age, education and sex as covariates. A chi‐square test was employed for the between‐group comparison on sex, followed by standardized adjusted residual‐based a posteriori decompositions (with cells yielding a *z*‐transformed residual ≥|2.87|, i.e. the critical value associated with the Bonferroni‐adjusted significance level, being deemed as significantly contributing to the *omnibus* effects). # = vs healthy controls; § = vs bvFTD;° = vs sbvFTD; $ = vs nfvPPA; *α* = vs svPPA; *vs PSP.

Abbreviations: 3FT, Three Faces Test; AD, affect discrimination; ARQ, affect recognition quotient; bvFTD, behavioural variant of frontotemporal dementia; CaGi, Catricalà‐Ginex Battery; CATS‐A, Abbreviated version of the Comprehensive Affect Test System; CDT, Clock Drawing Test; CI, causal inference; EA, emotion attribution; FAB, Frontal Assessment Battery; FBI, Frontal Behavioural Inventory; HC, healthy controls; IA, intention attribution; ID, identity discrimination; MA, match affect; MCST, Modified Card Sorting Test; MMSE, Mini‐Mental State Examination; NA, name affect; nfvPPA, nonfluent variant of primary progressive aphasia; PSP, progressive supranuclear palsy; PPT, Pyramid and Palm Trees Test; RAVLT, Rey Auditory Verbal Learning Test; RCPM, Raven's Colored Progressive Matrices; ROCF, Rey–Osterrieth Complex Figure; SA, select affect; sbvFTD, semantic behavioural variant of frontotemporal dementia; SET, Story‐Based Empathy Task; svPPA, semantic variant of primary progressive aphasia; TMT, Trail Making Test; WPM, Word Picture Matching.

^a^ No significant post hoc comparisons.

PSP cases were older than the other groups except for nfvPPA. BvFTD and PSP patients had lower education than controls. Nineteen bvFTD (4 C9orf72, 2 MAPT, 1 C9orf72 + MAPT, 1 FUS, 9 GRN, 1 TREM2), two sbvFTD (1 MAPT, 1 GRN), three nfvPPA (2 GRN), two svPPA (1 MAPT, 1 GRN) and one PSP (1 GRN) cases had FTD‐related genetic mutations (g‐FTD). Among those cases with available CSF, none presented with an Alzheimer's disease profile.

Compared with controls, all patients performed worse in verbal and visuospatial memory, abstract reasoning, phonemic and semantic fluency, verbal comprehension and AR. Additionally, all patients except for the sbvFTD group, performed worse than controls in global cognition, verbal working memory and selective attention. Compared with controls, only bvFTD and PSP cases performed worse in attention shifting, facial identification (CATS‐A ID) and complex figure copying, with bvFTD patients also performing worse in problem solving. Compared with controls, g‐FTD and g‐bvFTD cases showed lower performances in all CATS‐A subtests (Table S1).

Patient groups were similar in disease duration, executive dysfunctions (FAB), theory of mind, visuospatial abilities and behaviour. In this latter domain, bvFTD showed more behavioural disturbances compared with nfvPPA cases only. In general, compared with the other cases, bvFTD and PSP exhibited the poorest cognitive performance, whereas sbvFTD patients showed the best performance. The svPPA and sbvFTD groups performed similarly in naming, single‐word comprehension and object knowledge, but in these domains, only svPPA performed worse than nfvPPA patients. Genetic and sporadic cases performed similarly in all AR subtests, except for CATS‐A AD where g‐FTD cohort performed worse than s‐FTD (Table S2).

In patients, CATS‐A subscores proved to be underpinned by a simple, mono‐component structure (51.14% of variance explained), with all subtests substantially loading on the component itself (*range* = 0.61–0.80), as well as to be internally reliable (McDonald's ω = 0.76). Regardless of executive and comprehension subjects' abilities (i.e. FAB and token test scores), the CATS‐A ARQ was associated with the SET global score (*r*(101) = 0.28; *p* = 0.004).

### Affect recognition disease‐specific cut‐offs and diagnostics

The norming procedure in HC showed that the cubic transform of age was the sole predictor of CATS‐A ID scores (Table 2). CATS‐A AD, MA and ARQ adjustment equations have been previously reported [28].

**TABLE 2:** Adjustment equations for selected CATS‐A measures.

	Adjustment equation
CATS‐A ID	*Adjusted score* = *raw score*+0.000002*((age [3])‐270,028)

Abbreviations: CATS‐A, Abbreviated version of Comprehensive Affect Test System; ID, identity discrimination.

**FIGURE 1:**
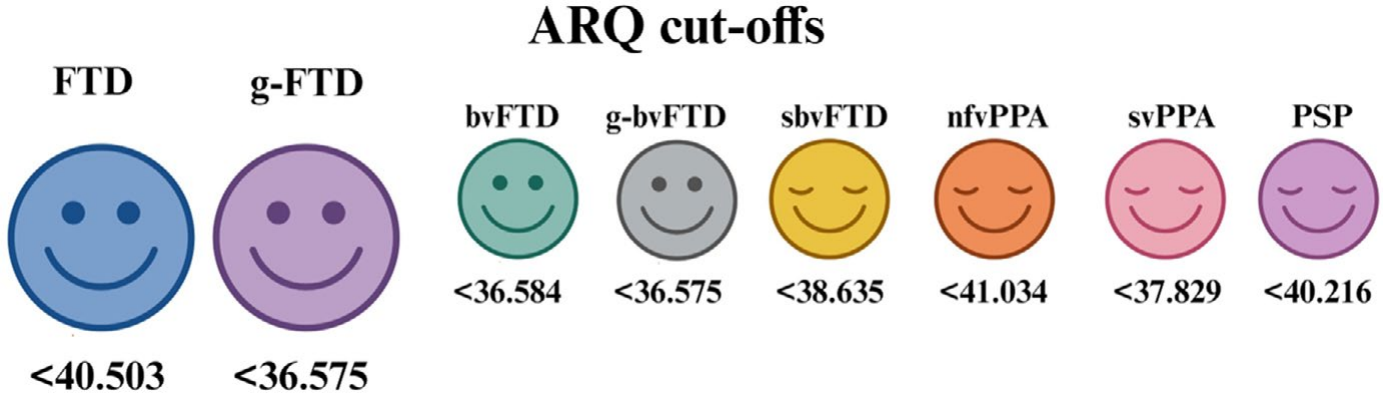
Disease‐specific cut‐offs for the CATS‐A ARQ. Colours represent the different clinical phenotypes. Happy faces (smile and open eyes) reflect a ‘good’ diagnostic performance (SUI≥1.28); content faces (smile and closed eyes) reflect an ‘adequate’ diagnostic performance (SUI≥0.98). Figure created with BioRender.com. ARQ, affect recognition quotient; bvFTD, behavioural variant of frontotemporal dementia; CATS‐A, Abbreviated version of Comprehensive Affect Test System; FTD, frontotemporal dementia; g‐bvFTD, patients with behavioural variant of frontotemporal dementia carrying genetic mutations; g‐FTD, patients with frontotemporal degeneration carrying genetic mutations; nfvPPA, nonfluent variant of primary progressive aphasia; sbvFTD, semantic behavioural variant of frontotemporal dementia; svPPA, semantic variant of primary progressive aphasia; PSP, progressive supranuclear palsy; SUI, Summary Utility Index.

All CATS‐A measures achieved acceptable accuracy in distinguishing HC from both the entire FTD cohort and each patient subgroup, except for CATS‐A AD in sbvFTD and svPPA, and CATS‐A ID in sbvFTD, svPPA and nfvPPA cases (Table 3). Cut‐offs and diagnostic metrics computed for CATS‐A measures featured by an AUC value ≥0.70 are displayed in Table 4 and Figure 1. According to SUI values, the ARQ systematically proved to be characterized by optimal diagnostic performances, both with regard to the whole FTD cohort and for each patient's clinical syndrome. As to CATS‐A subscores, their diagnostic performance was found to be adequate/good in the context of the discrimination between HC and both the whole patient cohort and bvFTD patients, whilst yielding heterogeneous findings in respect to the other syndromes. More specifically, the CATS‐A SA showed consistently optimal diagnostic features across different syndromes, except for svPPA patients; the CATS‐A MA adequately discriminated HC from each patient group but from svPPA and PSP patients; and the CATS‐A 3FT showed an adequate diagnostic performance in nfvPPA cases (other than bvFTD patients) and low diagnostic performance in sbvFTD, PSP and svPPA patients. With regard to their ability to identify sbvFTD, nfvPPA, svPPA and PSP patients, CATS‐A NA measures did not reach acceptable diagnostic performances. Finally, optimal diagnostics were detected as to the control condition (i.e. the CATS‐A ID) in the aim of discriminating HC from bvFTD and PSP patients, as well as from the whole FTD cohort.

**TABLE 3:** AUC values for CATS‐A measures (each patient group vs. healthy controls).

	AUC	*SE*	CI 95%
bvFTD			
CATS‐A ID	0.85	0.04	[0.78, 0.92]
CATS‐A AD	0.78	0.04	[0.70, 0.85]
CATS‐A NA	0.85	0.03	[0.78, 0.91]
CATS‐A SA	0.88	0.03	[0.82, 0.94]
CATS‐A MA	0.82	0.03	[0.76, 0.88]
CATS‐A 3FT	0.82	0.03	[0.76, 0.89]
CATS‐A ARQ	0.91	0.03	[0.86, 0.96]
sbvFTD			
CATS‐A ID	0.60	0.09	[0.42, 0.77]
CATS‐A AD	0.45	0.09	[0.28, 0.62]
CATS‐A NA	0.86	0.05	[0.76, 0.96]
CATS‐A SA	0.96	0.02	[0.92, 1]
CATS‐A MA	0.88	0.04	[0.80, 0.97]
CATS‐A 3FT	0.81	0.06	[0.69, 0.93]
CATS‐A ARQ	0.92	0.03	[0.86, 0.98]
nfvPPA			
CATS‐A ID	0.62	0.08	[0.46, 0.78]
CATS‐A AD	0.71	0.08	[0.56, 0.86]
CATS‐A NA	0.77	0.07	[0.64, 0.90]
CATS‐A SA	0.77	0.07	[0.63, 0.92]
CATS‐A MA	0.85	0.04	[0.77, 0.94]
CATS‐A 3FT	0.89	0.04	[0.82, 0.96]
CATS‐A ARQ	0.92	0.03	[0.86, 0.98]
svPPA			
CATS‐A ID	0.69	0.06	[0.56, 0.83]
CATS‐A AD	0.60	0.08	[0.45, 0.75]
CATS‐A NA	0.76	0.07	[0.64, 0.89]
CATS‐A SA	0.80	0.06	[0.69, 0.92]
CATS‐A MA	0.73	0.06	[0.61, 0.84]
CATS‐A 3FT	0.72	0.06	[0.61, 0.83]
CATS‐A ARQ	0.83	0.05	[0.73, 0.93]
PSP			
CATS‐A ID	0.76	0.06	[0.64, 0.88]
CATS‐A AD	0.70	0.06	[0.59, 0.82]
CATS‐A NA	0.84	0.04	[0.75, 0.92]
CATS‐A SA	0.79	0.06	[0.67, 0.91]
CATS‐A MA	0.75	0.05	[0.65, 0.86]
CATS‐A 3FT	0.76	0.05	[0.67, 0.86]
CATS‐A ARQ	0.85	0.05	[0.76, 0.94]
Whole FTD			
CATS‐A ID	0.75	0.03	[0.70, 0.81]
CATS‐A AD	0.70	0.03	[0.63, 0.76]
CATS‐A NA	0.82	0.03	[0.77, 0.87]
CATS‐A SA	0.85	0.03	[0.80, 0.89]
CATS‐A MA	0.80	0.03	[0.75, 0.86]
CATS‐A 3FT	0.80	0.03	[0.75, 0.86]
CATS‐A ARQ	0.89	0.02	[0.85, 0.93]

Abbreviations: 3FT, Three Faces Test; AD, affect discrimination; ARQ, affect recognition quotient; AUC, area under the curve; bvFTD, behavioural variant of frontotemporal dementia; CATS‐A, Abbreviated version of Comprehensive Affect Test System; CI, confidence interval; FTD, frontotemporal dementia; ID, identity discrimination; MA, match affect; NA, name affect; nfvPPA, nonfluent variant of primary progressive aphasia; PSP, progressive supranuclear palsy; SA, select affect; sbvFTD, semantic behavioural variant of frontotemporal dementia; SE, standard error; svPPA, semantic variant of primary progressive aphasia.

**TABLE 4:** Disease‐specific cut‐offs and diagnostic metrics for CATS‐A measures.

	Cut‐off	*J*	Se	Sp	PPV	NPV	LR+	LR−	SUI	Interpretation
bvFTD										
CATS‐A ID^a^	<10.538	0.65	0.70	0.95	0.88	0.86	13.53	0.32	1.43	Good
CATS‐A AD^a^	<10.131	0.48	0.63	0.85	0.68	0.82	4.98	0.43	1.13	Adequate
CATS‐A NA	≤4	0.55	0.92	0.63	0.56	0.94	2.47	0.13	1.11	Adequate
CATS‐A SA	≤4	0.65	0.73	0.91	0.82	0.87	8.51	0.29	1.39	Good
CATS‐A MA^a^	<8.41	0.48	0.82	0.66	0.56	0.88	2.43	0.28	1.04	Adequate
CATS‐A 3FT	≤13	0.50	0.93	0.57	0.53	0.94	2.17	0.12	1.03	Adequate
CATS‐A ARQ^a^	<36.584	0.70	0.78	0.91	0.83	0.89	9.09	0.24	1.46	Good
sbvFTD										
CATS‐A NA	≤4	0.55	0.92	0.63	0.22	0.99	2.49	0.12	0.83	Poor
CATS‐A SA	≤4	0.84	0.92	0.91	0.55	0.99	10.71	0.08	1.41	Good
CATS‐A MA^a^	<6.698	0.72	0.85	0.87	0.42	0.98	6.54	0.18	1.21	Adequate
CATS‐A 3FT	≤12	0.50	0.85	0.66	0.22	0.97	2.45	0.24	0.83	Poor
CATS‐A ARQ^a^	<38.635	0.73	0.92	0.81	0.35	0.99	4.87	0.1	1.12	Adequate
nfvPPA										
CATS‐A AD^a^	<10.410	0.43	0.65	0.78	0.31	0.94	3	0.45	0.93	Poor
CATS‐A NA	≤4	0.45	0.82	0.63	0.25	0.96	2.22	0.28	0.81	Poor
CATS‐A SA	≤4	0.5	0.59	0.91	0.5	0.94	6.82	0.45	1.15	Adequate
CATS‐A MA^a^	<7.614	0.57	0.82	0.75	0.33	0.97	3.29	0.24	1.00	Adequate
CATS‐A 3FT	≤11	0.62	0.88	0.74	0.33	0.98	3.41	0.16	1.02	Adequate
CATS‐A ARQ^a^	<41.034	0.68	0.94	0.74	0.35	0.99	3.64	0.08	1.06	Adequate
svPPA										
CATS‐A NA	≤4	0.44	0.81	0.63	0.28	0.95	2.18	0.30	0.83	Poor
CATS‐A SA	≤5	0.45	0.86	0.60	0.28	0.96	2.12	0.24	0.82	Poor
CATS‐A MA^a^	<8.092	0.39	0.71	0.67	0.28	0.93	2.18	0.43	0.82	Poor
CATS‐A 3FT	≤13	0.38	0.81	0.57	0.25	0.94	1.88	0.34	0.74	Poor
CATS‐A ARQ^a^	<37.829	0.54	0.67	0.87	0.48	0.94	5.16	0.38	1.14	Adequate
PSP										
CATS‐A ID^a^	<10.013	0.52	0.54	0.98	0.88	0.89	31.07	0.47	1.35	Good
CATS‐A AD^a^	<10.636	0.38	0.61	0.77	0.38	0.89	2.61	0.51	0.92	Poor
CATS‐A NA	≤4	0.56	0.93	0.63	0.38	0.97	2.51	0.11	0.96	Poor
CATS‐A SA	≤4	0.59	0.68	0.91	0.66	0.92	7.87	0.35	1.29	Good
CATS‐A MA^a^	<8.311	0.46	0.79	0.67	0.37	0.93	2.4	0.32	0.92	Poor
CATS‐A 3FT	≤13	0.46	0.89	0.57	0.33	0.96	2.07	0.19	0.84	Poor
CATS‐A ARQ^a^	<40.216	0.62	0.86	0.77	0.47	0.96	3.68	0.19	1.14	Adequate
Whole FTD										
CATS‐A ID^a^	<10.636	0.46	0.51	0.95	0.92	0.62	9.88	0.52	1.09	Adequate
CATS‐A AD^a^	<10.131	0.38	0.53	0.85	0.80	0.60	3.43	0.55	0.93	Poor
CATS‐A NA	≤4	0.52	0.89	0.63	0.74	0.83	2.41	0.17	1.18	Adequate
CATS‐A SA	≤4	0.61	0.69	0.91	0.91	0.71	8.01	0.34	1.27	Adequate
CATS‐A MA^a^	<8.311	0.47	0.80	0.67	0.75	0.74	2.44	0.3	1.10	Adequate
CATS‐A 3FT	≤13	0.48	0.91	0.57	0.72	0.84	2.1	0.16	1.13	Adequate
CATS‐A ARQ^a^	<40.503	0.64	0.87	0.77	0.82	0.83	3.74	0.17	1.35	Good

*Note*: Summary Utility Index (SUI) was computed as the following: (Se*PPV) + (Sp*NPV); with values <0.97 suggesting an overall ‘poor’ diagnostic performance, values ≥0.98 suggesting an overall ‘adequate’ diagnostic performance, and values ≥1.28 suggesting an overall ‘good’ diagnostic performance.

Abbreviations: 3FT, Three Faces Test; ARQ, affect recognition quotient; bvFTD, behavioural variant of frontotemporal dementia; CATS‐A, Abbreviated version of Comprehensive Affect Test System; FTD, frontotemporal dementia; ID, identity discrimination; LR+, positive likelihood ratio; LR−, negative likelihood ratio; MA, match affect; nfvPPA, nonfluent variant of primary progressive aphasia; NPV, negative predictive value; PPV, positive predictive value; PSP, progressive supranuclear palsy; SA, select affect; sbvFTD, semantic behavioural variant of frontotemporal dementia; Se, sensitivity; Sp, specificity; svPPA, semantic variant of primary progressive aphasia; SUI, Summary Utility Index.

^a^ These metrics are referred to demographically adjusted CATS‐A scores.

In discriminating HC from g‐FTD and from g‐bvFTD, all CATS‐A measures reached acceptable accuracy (Table S3). Cut‐offs and diagnostic metrics computed for CATS‐A measures featured by an AUC value ≥0.70 are displayed in Table S4. According to SUI values, the ARQ systematically proved to be characterized by optimal diagnostic performances, both with regard to the whole g‐FTD cohort and to the g‐bvFTD group. As to CATS‐A subscores, their diagnostic performance was found to be adequate/good in the context of the discrimination between HC and both the whole g‐FTD cohort and g‐bvFTD patients, except for CATS‐A NA and 3FT.

### Case–case discrimination

With regard to CATS‐A subscores, the CATS‐A ID, AD and 3FT were able to discriminate among different FTD phenotypes (CATS‐A AD: *F*(134, 4) = 2.71; *p* = 0.033; η^2^ = 0.08; CATS‐A 3FT: *F*(134, 4) = 4.07; *p* = 0.004; η^2^ = 0.11; CATS‐A ID: *F*(134, 4) = 3.63; *p* = 0.008; η^2^ = 0.10), whilst remaining ones were not (CATS‐A MA: *F*(134, 4) = 1.97; *p* = 0.103; η^2^ = 0.06; CATS‐A NA: *F*(134, 4) = 0.381; *p* = 0.822; η^2^ = 0.01; CATS‐A SA: *F*(134, 4) = 1.61; *p* = 175; η^2^ = 0.05). A posteriori comparisons revealed that bvFTD patients performed worse than sbvFTD on both the CATS‐A ID (*p* = 0.027; sbvFTD: *M* = 11.22, *SE* = 0.60; bvFTD: *M* = 9.18, *SE* = 0.44) and the CATS‐A AD (*p* = 0.027; sbvFTD: *M* = 11.26, *SE* = 0.54; bvFTD: *M* = 9.44, *SE* = 0.25), as well as that nfvPPA patients performed worse than both svPPA (*p* = 0.003) and PSP (*p* = 0.022) patients on the CATS‐A 3FT (nfvPPA: *M* = 8.00, *SE* = 0.71; svPPA: *M* = 11.5, *SE* = 0.63; PSP: *M* = 10.79, *SE* = 0.55).

As to the ARQ, whilst an *omnibus* effect of *Group* was detected (*F*(134, 4) = 2.82; *p* = 0.027; η^2^ = 0.08) Bonferroni‐corrected post hoc comparisons did not reveal any significant between‐group differences.

In discriminating patient groups (bvFTD vs sbvFTD; nfvPPA vs svPPA; nfvPPA vs PSP) that showed significant differences in some CATS‐A subtests (CATS‐A ID, AD and 3FT), all CATS‐A measures reached acceptable accuracy (AUC value ≥0.70; Table S5). Cut‐offs and diagnostic metrics computed for those CATS‐A measures are displayed in Table S6. According to SUI values, these subtests proved to be characterized by adequate diagnostic performances.

With regard to CATS‐A differences between genetic and sporadic cases, only the CATS‐A AD revealed to have lower scores in g‐FTD than s‐FTD patients (*p* = 0.023). However, this subtest did not reach acceptable accuracy (AUC = 0.66; SE = 0.06; CI 95% [0.55, 0.77]) in distinguishing g‐FTD patients from s‐FTD. Therefore, cut‐offs and diagnostic metrics were not computed.

## DISCUSSION

In this study, we established CATS‐A subscore cut‐offs for an Italian population of FTD patients, which can distinguish each FTD clinical syndrome from HC with high accuracy. We demonstrated that this battery is a powerful tool for detecting changes at the time of FTD diagnosis, serving as a marker of the disease. Notably, the ARQ consistently showed optimal diagnostic performance across the entire FTD cohort and within each patient group (range AUC = 0.83–0.92), highlighting the utility of administering the complete CATS‐A AR facial battery for detecting social perception changes in this clinical population. By demonstrating its applicability in a large sample of FTD (*N* = 139 cases), this study holds significant value for its clinical applications. Additionally, we provided optimal CATS‐A cut‐off values for specifically distinguishing g‐FTD and g‐bvFTD cases from HC. These reference values closely matched those observed in the entire cohort, though CATS‐A ARQ cut‐off was lower when distinguishing g‐FTD from HC (see Figure 1), likely reflecting a more severe AR involvement in these patients [31].

Disturbances in social cognition, particularly in emotion recognition, may contribute to personality and behaviour changes observed in FTD [32]. Understanding the impairment in emotion recognition across FTD subtypes will provide insights into the deficits experienced in these clinical syndromes, enhancing clinical diagnosis and management. Finally, the publication of cut‐offs and diagnostic accuracy for the Italian version is also relevant to a broader, non‐Italian audience. Specifically, four of the subitems (CATS‐A ID, AD, MA and 3FT) do not rely on verbal mediation, making it reasonable to assume that the cut‐offs and performance could be generalized across different languages.

In FTD cases, we found that CATS‐A ARQ correlated with scores from other measures of social cognition, such as the SET, an Italian paradigm examining affective and cognitive theory of mind [33]. Although significant, this correlation is not strong (*r* = 0.28). This can be explained by the fact that, whilst related, the two tests assess different dimensions of social cognition: CATS‐A evaluates affective recognition, whilst SET measures the ability of understanding others' intentions and emotional reactions. However, this relationship was not influenced by the presence of executive dysfunctions and language comprehension disturbances, which are symptoms typically exhibited by FTD phenotypes and that were also observed in this specific population. This is a crucial point that emphasizes the convergent validity of this battery and its distinctiveness from other cognitive domains. The question of whether AR performance is influenced by executive functions or operates independently from these cognitive processes remains a subject of debate [34, 35]. To date, several studies indicate early deficits in social cognition even before executive dysfunctions become apparent in FTD patients, such as bvFTD [5] and sbvFTD [36], suggesting a unique and early involvement of social cognitive domain in these conditions. In addition to executive dysfunction, language disturbances must also be taken into account to ensure adequate comprehension and production abilities during these tasks. Similar to the SET, CATS‐A addresses this issue by minimizing the use of verbal stimuli and instead instructing patients to point to the correct picture(s) in response to straightforward requests. Indeed, the influence of language appears to be minimal, at least in the case of ARQ scores.

Importantly, when considering CATS‐A subscores, their diagnostic efficacy was found to be adequate for distinguishing the entire patient cohort and bvFTD patients from HCs, but they produced heterogeneous results among other clinical syndromes. Specifically, CATS‐A SA consistently exhibited optimal diagnostic characteristics across different syndromes as compared with HCs, except for svPPA patients. In contrast, CATS‐A NA measures did not achieve acceptable diagnostic performance for identifying sbvFTD, nfvPPA, svPPA and PSP patients when compared to HCs, nor for distinguishing g‐FTD and g‐bvFTD from HCs. These subtests are the only ones that rely on verbal stimuli, specifically verbal labels for the six basic emotions plus neutral status (joy, fear, disgust, anger, surprise, sadness and neutral). This aspect could specifically affect the performance of patients with language comprehension issues, such as those with PPA, sbvFTD or genetic mutations.

Regarding the accuracy in distinguishing between groups of patients, we further observed that the CATS‐A ID, AD and 3FT subtests could discriminate among different FTD phenotypes, whilst the remaining subtests could not. Post hoc comparisons revealed that bvFTD patients performed worse than sbvFTD patients on both the CATS‐A ID and AD, and that nfvPPA patients performed worse than both svPPA and PSP patients on the CATS‐A 3FT. Furthermore, according to SUI values, these subtests proved to be characterized by adequate diagnostic performances (see Table S6 for cut‐offs).

Concerning the cognitive functioning of our FTD cases, bvFTD and sbvFTD patients exhibited performances at opposite extremes, with bvFTD performing the worst and sbvFTD performing the best in almost all investigated domains (except for the semantic domain). Therefore, the superiority of sbvFTD in the simpler facial subtests of CATS‐A was expected. Specifically, CATS‐A ID is the only subtest we used that investigated identity, rather than affect, discrimination. This subtest assesses the ability to extract invariant facial features (i.e. facial identity), regardless of changeable facial information (e.g. emotional expression, age and lip movements during speech) [2]. This ability is supported by the inferotemporal cortex, including the lateral fusiform face area [2]. Failures in identity discrimination have already been reported in bvFTD due to the involvement of these regions over the disease course [37]. On the contrary, the identification of invariant facial features is not impaired in sbvFTD. Instead, in these latter cases, impaired recognition of familiar or famous faces (i.e. prosopagnosia) has been constantly reported [21, 36, 38]. This is a very important finding in the field, as it demonstrates that CATS‐A ID and AD can effectively distinguish between bvFTD and sbvFTD cases.

The poorer performance of nfvPPA patients on the CATS‐A 3FT compared with svPPA and PSP patients is less clear. The impairment observed in this study is unlikely to be due to greater disease severity in the selected nfvPPA patients, as these participants had similar cognitive performance and disease duration to the other patient groups. However, a study involving nfvPPA cases suggested that increasing the salience of emotions, thereby reducing the attentional and perceptual demands of the task, led to improved performance in this group [32]. The authors suggested that emotion recognition disturbances in nfvPPA may be partly attributable to attentional deficits, especially in more challenging tests like the CATS‐A 3FT, where the emotional intensity is not modulated. This phenomenon could be due to a failure in the top‐down process, specifically the preferential allocation of spatial attention to emotional stimuli [32]. This process is mediated by the prefrontal attentional network, which is more compromised in nfvPPA compared at least to svPPA cases. This latter condition involves more bottom‐up, pre‐attentive processing in regions ranging from limbic/subcortical to cortical areas. Another hypothesis, which does not exclude the previous one, is that the decision‐making process during the CATS‐A 3FT is inherently more complex. Specifically, the task of comparing pairwise similarities may require greater selection and decision‐making abilities compared with the other assessments. In any case, the role of language comprehension demands in nfvPPA cannot be entirely ruled out.

Negative findings, such as the inability of some CATS‐A subtests in discriminating among different phenotypes could also indicate that subtests like the CATS‐A MA and the ARQ global score are uniformly distributed across the entire FTD spectrum, serving as common markers of frontotemporal degeneration rather than distinguishing specific phenotypes.

In the sample of HCs, we observed that age predicted CATS‐A ID. The relationship between advancing age and cognitive decline is well‐documented in the literature and here is expected given the overlap between brain structures involved in facial and emotional perception, such as the anterior cingulate, prefrontal regions and insula, and those known to decline with age [39]. However, a study investigating the effect of age on CATS‐A performance found that age was not significantly associated with a decline in CATS‐A facial task performance [40]. Nevertheless, there was a significant age effect when discrete emotions were examined, with negative emotions being more affected than positive ones [40]. The discrepancies between our findings and previous research could be due to the larger population in our study (*N* = 116) compared with the earlier one (*N* = 60). Similarly, in our recently published work [28], we found an effect of education on more complex CATS‐A subtests (such as CATS‐A MA) that was not observed previously. This inconsistency might be attributable to the educational level differences between the Italian and US samples of HCs, with the latter being highly educated and having all IQ means falling in the high average range.

Some limitations should be acknowledged in relation to this study. Firstly, despite our sample being relatively large, the sample size may still be limited for the purpose of defining cut‐offs. Secondly, we focused solely on facial AR and did not investigate prosody recognition in CATS‐A. Thirdly, the potential impact of language comprehension on certain CATS‐A subtests has not been entirely ruled out.

In conclusion, this study establishes specific cut‐offs advantageous for the Italian FTD population to detect social perception impairments, particularly emotional recognition dysfunctions. These cut‐offs are valuable in clinical practice for identifying alterations in these patients as markers of frontotemporal degeneration. Specifically, the CATS‐A ARQ is highly effective in distinguishing FTD patients and controls, making it an excellent tool for immediate use in clinical practice. Future studies are needed to determine how well these or other specific cut‐offs can distinguish FTD from Alzheimer's disease, in order to provide useful data for differential diagnosis, prognosis, treatments and precise inclusion in the available clinical trials.

## AUTHOR CONTRIBUTIONS

**Elisa Canu:** Conceptualization; data curation; investigation; project administration; resources; supervision; validation; visualization; writing – original draft; writing – review and editing. **Veronica Castelnovo:** Data curation; formal analysis; investigation; resources; visualization; writing – review and editing. **Edoardo Nicolò Aiello:** Formal analysis; investigation; methodology; resources; software; validation; visualization; writing – original draft; writing – review and editing. **Giulia De Luca:** Formal analysis; visualization; writing – review and editing. **Elisa Sibilla:** Data curation; resources; writing – review and editing. **Fabiola Freri:** Data curation; resources; writing – review and editing. **Chiara Tripodi:** Data curation; resources; writing – review and editing. **Edoardo Gioele Spinelli:** Data curation; resources; funding acquisition; writing – review and editing. **Giordano Cecchetti:** Data curation; resources; writing – review and editing. **Giuseppe Magnani:** Data curation; resources; writing – review and editing. **Francesca Caso:** Data curation; resources; writing – review and editing. **Paola Caroppo:** Data curation; resources; writing – review and editing. **Sara Prioni:** Data curation; resources; writing – review and editing. **Cristina Villa:** Data curation; resources; writing – review and editing. **Lucio Tremolizzo:** Data curation; resources; writing – review and editing. **Ildebrando Appollonio:** Data curation; resources; writing – review and editing. **Federico Verde:** Data curation; resources; writing – review and editing. **Nicola Ticozzi:** Data curation; resources; writing – review and editing. **Vincenzo Silani:** Data curation; resources; writing – review and editing. **Virginia E. Sturm:** Data curation; resources; writing – review and editing. **Katherine P. Rankin:** Data curation; resources; writing – review and editing. **Maria Luisa Gorno‐Tempini:** Conceptualization; data curation; resources; writing – review and editing. **Barbara Poletti:** Conceptualization; data curation; resources; writing – review and editing; supervision; visualization. **Massimo Filippi:** Conceptualization; data curation; funding acquisition; project administration; resources; visualization; writing – review and editing. **Federica Agosta:** Conceptualization; data curation; resources; funding acquisition; project administration; supervision; visualization; writing – review and editing.

## FUNDING INFORMATION

This study was supported by European Research Council (StG‐2016_714388_NeuroTRACK) and the Foundation Research on Alzheimer Disease (France). We also acknowledge co‐funding from Next Generation EU, in the context of the National Recovery and Resilience Plan, Investment PE8—Project Age‐It: ‘Ageing Well in an Ageing Society’. Edoardo G Spinelli was co‐financed by the Next Generation EU (DM 1557 11.10.2022). The views and opinions expressed are only those of the authors and do not necessarily reflect those of the European Union or the European Commission. Neither the European Union nor the European Commission can be held responsible for them.

## CONFLICT OF INTEREST STATEMENT

E. Canu has received research supports from the Italian Ministry of Health. F. Verde is an associate editor for Journal of Alzheimer's Disease. N. Ticozzi received compensation for consulting services from Amylyx Pharmaceuticals and Zambon Biotech SA, and he is an associate editor for Frontiers in Aging Neuroscience. V. Silani received compensation for consulting services and/or speaking activities from AveXis, Cytokinetics, Italfarmaco, Liquidweb S.r.l., Novartis Pharma AG, Amylyx Pharmaceuticals, Biogen and Zambon Biotech SA, he receives or has received research supports from the Italian Ministry of Health, AriSLA and E‐Rare Joint Transnational Call, and he is in the Editorial Board of Amyotrophic Lateral Sclerosis and Frontotemporal Degeneration, European Neurology, American Journal of Neurodegenerative Diseases, Frontiers in Neurology, and Exploration of Neuroprotective Therapy. B. Poletti received compensation for consulting services and/or speaking activities from Liquidweb S.r.l, and she is an associate editor for Frontiers in Neuroscience. M. Filippi is Editor‐in‐Chief of the Journal of Neurology and an associate editor of Human Brain Mapping, Neurological Sciences, and Radiology; he received compensation for consulting services from Alexion, Almirall, Biogen, Merck, Novartis, Roche, Sanofi, speaking activities from Bayer, Biogen, Celgene, Chiesi Italia SpA, Eli Lilly, Genzyme, Janssen, Merck‐Serono, Neopharmed Gentili, Novartis, Novo Nordisk, Roche, Sanofi, Takeda and TEVA, participated in Advisory Boards for Alexion, Biogen, Bristol‐Myers Squibb, Merck, Novartis, Roche, Sanofi, Sanofi‐Aventis, Sanofi‐Genzyme and Takeda, and done scientific direction of educational events for Biogen, Merck, Roche, Celgene, Bristol‐Myers Squibb, Lilly, Novartis and Sanofi‐Genzyme; he receives research support from Biogen Idec, Merck‐Serono, Novartis, Roche, the Italian Ministry of Health, the Italian Ministry of University and Research and Fondazione Italiana Sclerosi Multipla. F. Agosta is an associate editor of NeuroImage: Clinical, has received speaker honoraria from Biogen Idec, Italfarmaco, Roche, Zambon and Eli Lilly, and received or has received research supports from the Italian Ministry of Health, the Italian Ministry of University and Research, AriSLA (Fondazione Italiana di Ricerca per la SLA), the European Research Council, the EU Joint Programme—Neurodegenerative Disease Research (JPND) and Foundation Research on Alzheimer Disease (France). V. Castelnovo, E.N. Aiello, G. de Luca, E. Sibilla, F. Freri, C. Tripodi, E.G. Spinelli, G. Cecchetti, G. Magnani, F. Caso, P. Caroppo, S. Prioni, C. Villa, L. Tremolizzo, I. Appollonio, V. Sturm, K.P. Rankin and M.L. Gorno‐Tempini have nothing to disclose.

## Supporting information


Appendix S1.

## Data Availability

The dataset used and analysed during the current study will be made available by the corresponding author upon request to qualified researchers (i.e. affiliated to a university or research institution/hospital).
