# Supplementary material for: A common marker of affect recognition dysfunction in the FTD spectrum of disorders
Source: Eur J Neurol. 2024 Dec 4;32(1):e16578. doi: 10.1111/ene.16578 (PMC11617592; doi:10.1111/ene.16578)
Supplement: Supplementary file 1 — Appendix S1. [file ENE-32-e16578-s001.docx]

**Supplementary material**

**Cognitive and behavioural assessment**

Neuropsychological assessments were performed by experienced neuropsychologists. In all patients, AR was evaluated using the CATS-A,^1^ which investigates different aspects of emotion processing using the Ekman pictures of facial affect, which express the six basic emotions (disgust, surprise, happiness, anger, fear and sadness). From this battery, we selected and administered the following subtests: identity discrimination (ID; 12 trials in which the patient is required to state whether two presented faces represent the same or different persons); affect discrimination (AD; 12 trials in which the patient is required to state whether two presented faces express the same or different emotions); name affect (NA; 6 trials in which the patient is required to select, among 7 possible choices, the emotional label that best describes the emotion expressed by the face target); select affect (SA; 6 trials in which the patient is required to select, among 5 possible facial affect pictures, which is the one expressing the labeled emotion); match affect (MA; 12 trials in which the patient is required to select, among 5 possible facial affect pictures, which is the one expressing the same emotion of the face target); Three Faces Test (3FT; 12 trials in which the patient is required to select, among 3 possible facial affect pictures, which is the couple expressing the same emotion). We obtained specific scores (i.e., number of correct answers) for each CATS-A subdomain; furthermore, by summing the scores of all affect recognition subtests (all subtests except for CATS-A ID), we obtained a total score of affect recognition quotient (ARQ).

The following cognitive functions were also investigated, as previously described:^2^ global cognitive functioning with the MMSE^3^ and the frontal assessment battery (FAB);^4^ long and short term verbal memory with the Rey Auditory Verbal Learning Test (RAVLT)^5^ and digit span forward;^6^ long and short term spatial memory with the recall of the Rey’s complex figure (ROCF),^7^ and the spatial span forward;^6^ attention and executive functions with the digit span backward,^6^ the Modified Card Sorting Test (MCST),^8^ attentive matrices,^9^ trail making test (TMT),^10^ and the Raven’s coloured progressive matrices (RCPM);^11^ theory of mind with the Story-based Empathy Task (SET);^12^ language with the token test,^13^ semantic and phonemic fluency tests;^14^ visuospatial abilities with the ROCF copy,^7^ and the clock drawing test (CDT);^15^ the presence of behavioural disturbances with the frontal behavioural inventory (FBI)^16^ administered to patients’ caregivers. In addition, for sbvFTD and PPA patients, we investigated naming and single-word comprehension with the subtests of the CaGi battery,^17^ and object knowledge with the Pyramids and Palm Trees (PPT) test.^18^

Healthy controls underwent the same assessment of patients except for FAB and SET.

**Table S1.** Comparisons on CATS-A scores between patients carrying genetic mutations and HC.

|  | **HC** | **g-FTD** | **g-bvFTD** | **p**  **HC *vs* g-FTD** | **p**  **HC *vs***  **g-bvFTD** |
| --- | --- | --- | --- | --- | --- |
| **N** | 116 | 26 | 18 |  |  |
| **CATS-A ID*** | 11.65 ± 0.62  (8.41 – 12.00) | 9.30 ± 2.54  (4.70 – 12.00) | 8.86 ± 2.62  (4.70 – 12.00) | <.001 | <.001 |
| **CATS-A AD*** | 11.14 ± 1.00  (6.96 – 12.00) | 9.09 ± 2.02  (4.61 – 12.00) | 8.90 ± 1.98  (5.70 – 12.00) | <.001 | <.001 |
| **CATS-A NA** | 4.58 ± 1.09  (2 – 6) | 2.77 ± 1.73  (0 – 6) | 2.61 ± 1.42  (0 – 5) | <.001 | <.001 |
| **CATS-A SA** | 5.48 ± 0.73  (3 – 6) | 3.62 ± 1.68  (1 – 6) | 3.17 ± 1.65  (1 – 6) | <.001 | <.001 |
| **CATS-A MA*** | 8.79 ± 1.77  (3.82 – 12.00) | 6.17 ± 2.53  (1.59 – 11.82) | 5.92 ± 2.45  (1.59 – 9.81) | <.001 | <.001 |
| **CATS-A 3FT** | 13.87 ± 3.26  (6 – 22) | 10.04 ± 2.69  (5 – 14) | 9.94 ± 2.60  (5 – 14) | <.001 | <.001 |
| **CATS-A ARQ*** | 43.85 ± 5.34 (26.90 – 56.47) | 32.81 ± 8.29  (16.59 – 50.13) | 31.85 ± 8.46  (16.59 – 50.13) | <.001 | <.001 |

Abbreviations. AD= Affect Discrimination; ARQ= Affect Recognition Quotient; CATS-A=Abbreviated version of Comprehensive Affect Test System; CI=Confidence interval; ID=Identity Discrimination; FTLD=frontotemporal lobar degeneration; g-bvFTD= patients with behavioural variant of frontotemporal dementia and genetic mutations; g-FTD=patients with frontotemporal degeneration and genetic mutations; HC=healthy controls; MA= Match Affect; NA= Name Affect; 3FT= Three Faces Test. *P*-values refer to independent-sample *t-*tests. *Comparisons performed on demographically adjusted scores.

**Table S2.** Comparisons on CATS-A scores between sporadic FTD cases and patients carrying genetic mutations.

|  | **g-FTD** | **s-FTD** | ***p*** | **g-bvFTD** | **s-bvFTD** | ***p*** |
| --- | --- | --- | --- | --- | --- | --- |
| ***N*** | 26 | 113 |  | 18 | 42 |  |
| **CATS-A ID*** | 9.30 ± 2.54 (4.70 – 12.00) | 10.00 ± 2.17 (0.00 – 12.00) | .153 | 8.86 ± 2.62 (4.70 – 12.00) | 9.32 ± 1.97 (5.83 – 12.00) | .459 |
| **CATS-A AD*** | 9.09 ± 2.02 (4.61 – 12.00) | 10.07 ± 1.96 (0.00 – 12.00) | .023 | 8.90 ± 1.98 (5.70 – 12.00) | 9.68 ± 1.80 (5.83 – 12.00) | .141 |
| **CATS-A NA** | 2.77 ± 1.73  (0 – 6) | 2.84 ± 1.42  (0 – 6) | .825 | 2.61 ± 1.42  (0 – 5) | 2.71 ± 1.50  (0 – 6) | .805 |
| **CATS-A SA** | 3.62 ± 1.68  (1 – 6) | 3.75 ± 1.39  (0 – 6) | .665 | 3.17 ± 1.65  (1 – 6) | 3.64 ± 1.32  (0 – 6) | .241 |
| **CATS-A MA*** | 6.17 ± 2.53 (1.59 – 11.82) | 6.51 ± 2.06 (0.00 – 10.59) | .468 | 5.92 ± 2.45 (1.59 – 9.81) | 6.57 ± 1.70 (2.03 – 9.03) | .241 |
| **CATS-A 3FT** | 10.04 ± 2.69  (5 – 14) | 10.09 ± 3.12  (0 – 19) | .940 | 9.94 ± 2.6  (5 – 14) | 9.71 ± 3.01  (4 – 18) | .779 |
| **CATS-A ARQ*** | 32.81 ± 8.29 (16.59 – 50.13) | 33.73 ± 6.72 (0.00 – 51.84) | .547 | 31.85 ± 8.46 (16.59 – 50.13) | 32.62 ± 5.97 (21.59 – 47.47) | .693 |

Abbreviations. AD= Affect Discrimination; ARQ= Affect Recognition Quotient; CATS-A=Abbreviated version of Comprehensive Affect Test System; CI=Confidence interval; ID=Identity Discrimination; FTLD=frontotemporal lobar degeneration; g-bvFTD= patients with behavioural variant of frontotemporal dementia and genetic mutations; g-FTD=patients with frontotemporal degeneration and genetic mutations; HC=healthy controls; MA= Match Affect; NA= Name Affect; s-bvFTD= patients with sporadic behavioural variant of frontotemporal dementia; s-FTD=patients with sporadic frontotemporal degeneration; 3FT= Three Faces Test. *P*-values refer to independent-sample *t-*tests. *Comparisons performed on demographically adjusted scores.

**Table S3.** AUC values for CATS-A measures for the discrimination between HC from g-FTD and g-bvFTD.

|  | **AUC** | ***SE*** | **CI 95%** |
| --- | --- | --- | --- |
| **g-FTD** | | | |
| CATS-A ID | .76 | .07 | [.64, .89] |
| CATS-A AD | .83 | .05 | [.73, .93] |
| CATS-A NA | .80 | .06 | [.69, .91] |
| CATS-A SA | .82 | .06 | [.71, .93] |
| CATS-A MA | .80 | .05 | [.69, .91] |
| CATS-A 3FT | .81 | .04 | [.74, .89] |
| CATS-A ARQ | .87 | .05 | [.78, .97] |
| **g-bvFTD** | | | |
| CATS-A ID | .81 | .07 | [.67, .95] |
| CATS-A AD | .84 | .06 | [.72, .96] |
| CATS-A NA | .86 | .04 | [.78, .94] |
| CATS-A SA | .88 | .06 | [.77, .99] |
| CATS-A MA | .82 | .06 | [.71, .94] |
| CATS-A 3FT | .82 | .04 | [.74, .91] |
| CATS-A ARQ | .90 | .05 | [.80, .99] |

Abbreviations. AD= Affect Discrimination; ARQ= Affect Recognition Quotient; AUC=Area under the curve; bvFTD= behavioural variant of frontotemporal dementia; CATS-A=Abbreviated version of Comprehensive Affect Test System; CI=Confidence interval; ID=Identity Discrimination; FTLD=frontotemporal lobar degeneration; g-bvFTD= patients with behavioural variant of frontotemporal dementia and genetic mutations; g-FTD=patients with frontotemporal degeneration and genetic mutations; MA= Match Affect; NA= Name Affect; SE= Standard error; 3FT= Three Faces Test.

**Table S4.** Disease-specific cut-offs and diagnostic metrics for the discrimination between HC and g-FTD and g-bvFTD patients.

|  | **Cut-off** | ***J*** | **Se** | **Sp** | **PPV** | **NPV** | **LR+** | **LR-** | **SUI** | **Interpretation** |
| --- | --- | --- | --- | --- | --- | --- | --- | --- | --- | --- |
| **g-FTD** | | | | | | | | | | |
| CATS-A ID^1^ | <10.062 | .56 | .58 | .98 | .88 | .86 | 33.46 | .43 | 1.35 | Good |
| CATS-A AD^1^ | <10.103 | .62 | .77 | .85 | .54 | .94 | 5.25 | .27 | 1.21 | Adequate |
| CATS-A NA | ≤4 | .48 | .85 | .63 | .34 | .95 | 2.28 | .24 | .89 | Poor |
| CATS-A SA | ≤4 | .60 | .69 | .91 | .64 | .93 | 8.03 | .34 | 1.29 | Good |
| CATS-A MA^1^ | <6.812 | .48 | .65 | .85 | .49 | .92 | 4.21 | .41 | 1.10 | Adequate |
| CATS-A 3FT | ≤13 | .49 | .92 | .57 | .32 | .97 | 2.14 | .14 | .85 | Poor |
| CATS-A ARQ^1^ | <36.575 | .68 | .77 | .91 | .67 | .95 | 8.92 | .25 | 1.38 | Good |
| **g-bvFTD** | | | | | | | | | | |
| CATS-A ID^1^ | <10.062 | .65 | .67 | .98 | .86 | .95 | 38.67 | .34 | 1.51 | Good |
| CATS-A AD^1^ | <10.103 | .63 | .78 | .85 | .45 | .96 | 5.31 | .26 | 1.17 | Adequate |
| CATS-A NA | ≤4 | .57 | .94 | .63 | .28 | .99 | 2.55 | .09 | .89 | Poor |
| CATS-A SA | ≤4 | .69 | .78 | .91 | .58 | .96 | 9.02 | .24 | 1.33 | Good |
| CATS-A MA^1^ | <6.812 | .57 | .72 | .85 | .42 | .95 | 4.65 | .33 | 1.11 | Adequate |
| CATS-A 3FT | ≤13 | .51 | .94 | .57 | .25 | .99 | 2.19 | .10 | .89 | Poor |
| CATS-A ARQ^1^ | <36.575 | .73 | .83 | .91 | .60 | .97 | 9.67 | .18 | 1.38 | Good |

Abbreviations. ARQ= Affect Recognition Quotient; bvFTD= behavioural variant of frontotemporal dementia**;** CATS-A= Abbreviated version of Comprehensive Affect Test System; FTLD=frontotemporal lobar degeneration; ID=Identity Discrimination; g-bvFTD= patients with behavioural variant of frontotemporal dementia and genetic mutations; g-FTD=patients with frontotemporal degeneration and genetic mutations; LR+= positive likelihood ratio; LR-= negative likelihood ratio; MA= Match Affect; NPV=negative predictive value; PPV=positive predictive value; SA= Select Affect; Se= sensitivity; Sp= specificity; SUI=Summary Utility Index; 3FT= Three Faces Test. ^1^These metrics are referred to demographically adjusted CATS-A scores.

**Table S5.** AUC values for CATS-A measures within case-case discrimination analyses.

|  | **AUC** | ***SE*** | **CI 95%** |
| --- | --- | --- | --- |
| **bvFTD *vs* sbvFTD** | | | |
| CATS-A ID | .78 | .06 | [.65, .90] |
| CATS-A AD | .79 | .07 | [.66, .92] |
| **nfvPPA *vs* svPPA** | | | |
| CATS-A 3FT | .76 | .08 | [.62, .93] |
| **nfvPPA *vs* PSP** | | | |
| CATS-A 3FT | .72 | .08 | [.56, .88] |

Abbreviations. AD= Affect Discrimination; bvFTD= behavioural variant of frontotemporal dementia**;** CATS-A= Abbreviated version of Comprehensive Affect Test System; ID=Identity Discrimination; 3FT= Three Faces Test; nfvPPA=non-fluent variant-primary progressive aphasia; sbvFTD=semantic behavioural variant-frontotemporal dementia; svPPA=semantic variant-primary progressive aphasia; PSP=progressive supranuclear palsy.

**Table S6.** Disease-specific cut-offs and diagnostic metrics for CATS-A measures within case-case discrimination analyses.

|  | **Cut-off** | ***J*** | **Se** | **Sp** | **PPV** | **NPV** | **LR+** | **LR-** | **SUI** | **Interpretation** |
| --- | --- | --- | --- | --- | --- | --- | --- | --- | --- | --- |
| **bvFTD *vs* sbvFTD** | | | | | | | | | | |
| CATS-A ID^1^ | <10.583 | .55 | .70 | .85 | .96 | .38 | 4.55 | .36 | 1 | Adequate |
| CATS-A AD^1^ | <10.972 | .55 | .78 | .77 | .94 | .44 | 3.39 | .28 | 1.07 | Adequate |
| **nfvPPA *vs* svPPA** | | | | | | | | | | |
| CATS-A 3FT | ≤8 | .60 | .53 | .91 | .82 | .70 | 5.56 | .52 | 1.07 | Adequate |
| **nfvPPA *vs* PSP** | | | | | | | | | | |
| CATS-A 3FT | ≤6 | .31 | .35 | .96 | .86 | .71 | 9.88 | .67 | .98 | Adequate |

Abbreviations. AD= Affect Discrimination; bvFTD= behavioural variant of frontotemporal dementia; CATS-A= Abbreviated version of Comprehensive Affect Test System; ID=Identity Discrimination; LR+= positive likelihood ratio; LR-= negative likelihood ratio; NPV=negative predictive value; PPV=positive predictive value; SA= Select Affect; Se= sensitivity; Sp= specificity; SUI=Summary Utility Index; 3FT= Three Faces Test. ^1^These metrics are referred to demographically adjusted CATS-A scores.

**References**

1. Froming K, Levy M SS, Ekman P. The comprehensive affect testing system: Psychology Software, Inc, 2006.

2. Cividini C, Basaia S, Spinelli EG, et al. Amyotrophic Lateral Sclerosis-Frontotemporal Dementia: Shared and Divergent Neural Correlates Across the Clinical Spectrum. Neurology 2021.

3. Folstein MF, Folstein SE, McHugh PR. "Mini-mental state". A practical method for grading the cognitive state of patients for the clinician. J Psychiatr Res 1975;12:189-198.

4. Appollonio I, Leone M, Isella V, et al. The Frontal Assessment Battery (FAB): normative values in an Italian population sample. Neurol Sci 2005;26:108-116.

5. Carlesimo GA, Caltagirone C, Gainotti G. The Mental Deterioration Battery: normative data, diagnostic reliability and qualitative analyses of cognitive impairment. The Group for the Standardization of the Mental Deterioration Battery. Eur Neurol 1996;36:378-384.

6. Monaco M, Costa A, Caltagirone C, Carlesimo GA. Forward and backward span for verbal and visuo-spatial data: standardization and normative data from an Italian adult population. Neurol Sci 2013;34:749-754.

7. Caffarra P, Vezzadini G, Dieci F, Zonato F, Venneri A. Rey-Osterrieth complex figure: normative values in an Italian population sample. Neurol Sci 2002;22:443-447.

8. Caffarra P, Vezzadini G, Dieci F, Zonato F, Venneri A. Modified Card Sorting Test: normative data. J Clin Exp Neuropsychol 2004;26:246-250.

9. Spinnler H, Tognoni G. Standardizzazione e taratura italiana di test neuropsicologici. Ital J Neurol Sci 1987;6, suppl 8:44-46.

10. Giovagnoli AR, Del Pesce M, Mascheroni S, Simoncelli M, Laiacona M, Capitani E. Trail making test: normative values from 287 normal adult controls. Ital J Neurol Sci 1996;17:305-309.

11. Basso A, Capitani E, Laiacona M. Raven's coloured progressive matrices: normative values on 305 adult normal controls. Funct Neurol 1987;2:189-194.

12. Dodich A, Cerami C, Canessa N, et al. A novel task assessing intention and emotion attribution: Italian standardization and normative data of the Story-based Empathy Task. Neurol Sci 2015;36:1907-1912.

13. De Renzi E, Vignolo LA. The token test: A sensitive test to detect receptive disturbances in aphasics. Brain 1962;85:665-678.

14. Novelli G, Laiacona M, Papagno C, Vallar G, Capitani E, Cappa SF. Three clinical tests to research and rate the lexical performance of normal subjects. Arch Psicol Neurol Psichiatr 1986;47:477-506.

15. Manos PJ. Ten-point clock test sensitivity for Alzheimer's disease in patients with MMSE scores greater than 23. Int J Geriatr Psychiatry 1999;14:454-458.

16. Alberici A, Geroldi C, Cotelli M, et al. The Frontal Behavioural Inventory (Italian version) differentiates frontotemporal lobar degeneration variants from Alzheimer's disease. Neurol Sci 2007;28:80-86.

17. Catricala E, Della Rosa PA, Ginex V, Mussetti Z, Plebani V, Cappa SF. An Italian battery for the assessment of semantic memory disorders. Neurol Sci 2013;34:985-993.

18. Gamboz N, Coluccia E, Iavarone A, Brandimonte MA. Normative data for the Pyramids and Palm Trees Test in the elderly Italian population. Neurol Sci 2009;30:453-458.
